# The Self-Reported Oral Health Status and Dental Attendance of Smokers and Non-Smokers in England

**DOI:** 10.1371/journal.pone.0148700

**Published:** 2016-02-10

**Authors:** Julia Csikar, Jing Kang, Ceri Wyborn, Tom A. Dyer, Zoe Marshman, Jenny Godson

**Affiliations:** 1 School of Dentistry, University of Leeds, Leeds, LS2 9LU, United Kingdom; 2 Public Health England, Blenheim House, Leeds, LS1 4PL, United Kingdom; 3 Public Health England, West Offices, Station Rise, York, YO1 6GA, United Kingdom; 4 School of Clinical Dentistry, University of Sheffield, Claremont Crescent, Sheffield, S10 2TA, United Kingdom; University College London, UNITED KINGDOM

## Abstract

Smoking has been identified as the second greatest risk factor for global death and disability and has impacts on the oral cavity from aesthetic changes to fatal diseases such as oral cancer. The paper presents a secondary analysis of the National Adult Dental Health Survey (2009). The analysis used descriptive statistics, bivariate analyses and logistic regression models to report the self-reported oral health status and dental attendance of smokers and non-smokers in England. Of the 9,657 participants, 21% reported they were currently smoking. When compared with smokers; non-smokers were more likely to report ‘good oral health’ (75% versus 57% respectively, p<0.05). Smokers were twice as likely to attend the dentist symptomatically (OR = 2.27, CI = 2.02–2.55) compared with non-smoker regardless the deprivation status. Smokers were more likely to attend symptomatically in the most deprived quintiles (OR = 1.99, CI = 1.57–2.52) and perceive they had poorer oral health (OR = 1.77, CI = 1.42–2.20). The present research is consistent with earlier sub-national research and should be considered when planning early diagnosis and management strategies for smoking-related conditions, considering the potential impact dental teams might have on smoking rates.

## Introduction

Smoking (including second-hand smoke) has been identified as the second greatest risk factor for global death and disability [1]. Smoking increases the risk of heart disease, stroke, chronic lung disease and is the primary cause of cancer of the lungs, larynx, oesophagus, mouth, and bladder, and has been linked to cancer of the cervix, pancreas and kidneys [2, 3]. In England, 18% of all deaths (aged 35 years and over) were attributed directly to smoking [4].

The impacts from smoking on the oral cavity can include aesthetic changes such as stained teeth, discoloured 'tooth-coloured' restorations and dentures. There are also more serious complications related to smoking such as an increased prevalence of periodontitis leading to tooth loss, increased bone loss, impaired wound healing [5] and adverse effects on connective tissue repair [6]. The most serious condition associated with smoking and tobacco use is oral cancer [7–9]. The England mortality rate for oral cancer (The International Classification of Diseases (ICD10) used the following codes: C00-06, C09-10, C12-14) was 1,883 (per 100,000 population) per year in 2011 (males 1,221, females 662) [10]. The dental team have been suggested as having a key role in identifying smokers and tobacco users and providing information on reducing risks and also onward referral to smoking cessation services [11]. Referring to smoking cessations services [12] increases a smoker’s chance of successful cessation four-fold [13].

Analysis of data gathered in Yorkshire and the Humber [14] found links between smoking status and perceived oral health and dental attendance [15]. The analysis revealed that perceived oral health and dental attendance of smokers differed from non-smokers, irrespective of deprivation. For example, those who smoked were less likely to attend for routine dental check-ups and more likely to perceive they had poor oral health and to attend the dentist symptomatically. As smoking has multiple impacts on the oral cavity, dental teams have been targeted to deliver measures aimed at reducing tobacco use. It is therefore essential to understand if smokers access routine care differently to non-smokers and if smokers are a potentially ‘hard to reach’ group and how this may affect future policy recommendations development. A national dataset, The Adult Dental Health Survey (ADHS) 2009 [16], which sampled 13,400 households across England, Wales and Northern Ireland forms the basis of the present analysis. Thus, the aim of this paper is to report the self-reported oral health status and dental attendance of smokers and non-smokers in England.

## Materials and Methods

### Data

This was a secondary analysis of the National Adult Dental Health Survey (ADHS) (2009) [16], this de-annoymised data is openly available using an end user licence system (data can be obtained after registration: https://discover.ukdataservice.ac.uk/catalogue/?sn=6884&type=Data%20catalogue).

### Survey design

The ADHS sampled 13,400 households across England, Wales and Northern Ireland, using a two-stage cluster sampling technique (268 primary sampling units (PSU) across the UK, and each PSU consisted of two postcode sectors with 25 addresses sampled from each). This survey is representative at the level of each of the former Strategic Health Authority (SHA) regions in England. This paper will focus on the England data only (10 SHA, and 23 PSU in each SHA, so total 11,500 addresses were sampled) (adults aged 16 years and over). The Index of Multiple Deprivation for England 2010 was matched to each respondent’s data.

### Measures

The following measures were taken from the ADHS (2009) and are as follows:

- Age: 7 categories (16–24, 25–34, 35–44, 45–54, 55–64, 65–74, and 75 and over)
- Gender: male and female
- Ethnicity: 9 categories (White British/other White, mixed race, Asian-Indian, Asian-Pakistani & Bangladeshi, Asian-other, black Caribbean, black African, other black, and other ethnic group)
- Deprivation status: IMD was assigned to the participant’s postcode post interview (IMD for England (2010) [17]). Quintile 1 means most deprived, and quintile 5 means least deprived
- Smoking status (Yes or no): *‘do you smoke cigarettes at all nowadays*?’
- English regions: 10 regions (North East, North West, Yorkshire & the Humber, East Midlands, West Midlands, East of England, London, South East coast, South Central, and South West)
- Self-reported oral health status (good/poor): ‘*Would you say your dental health (mouth*, *teeth and/or dentures) is very good*, *good*, *fair*, *poor*, *or very poor*?’ For analysis purposes we classified ‘very good’ and ‘good’ as ‘good’, and ‘fair’, ‘poor’, or ‘very poor’ as ‘poor’
- Dental attendance: ‘*In general do you go to the dentist for a regular check-up*, *an occasional check-up*, *only when you're having trouble with your teeth/dentures*? *or never been to the dentist*’. Our analysis focused on ‘regular check-ups’ versus ‘symptomatic dental attendance’ (those who only attend dental practice when they are having trouble with their teeth or dentures).

### Analysis

The data analysis had three stages: firstly, descriptive statistics were used to understand the survey samples’ demographic features.

Secondly, bivariate analyses were undertaken using Chi-squared tests to investigate the associations between smoking and each demographic feature (such as participants’ gender, age, region and IMD). The associations between smoking and self-reported poor oral health and symptomatic dental attendance were investigated using Chi-square tests.

Finally, logistic regression models were applied to identify significant explanatory variables (age, sex, ethnicity, area, smoking status, and deprivation) related to poor self-reported oral health outcome and symptomatic dental attendance. The correlation of the variables or the multicollinearity within the model was assessed using the variance inflation factor (VIF) [18]. All statistical tests were performed at 0.05 significance level, using statistical software Rstudio with its Survey Package and SPSS 19. A survey weight was employed in both the logistic regression models to compensate for the differential sampling rates in each region and to reduce bias caused by non-response. Weighting information is presented in the extracted dataset which is derived from information of each PSU from 2001 Census (such as typical household type, social-economic status, ethnicity, age, sex) and each dentate adult (health status, dental behaviour, self-reported dental health and anxiety) [19].

## Results

### Demographic information

The ADHS dataset of English respondents equalled 9,663 adults, however due to IMD codes missing on 6 respondents these were removed from the dataset leaving 9,657 eligible to be included analysis (4,311 males: 44.6%) who completed a structured interview, 5,622 participants underwent a clinical dental examination.

The distribution of participants’ socio-demographic features are presented in Table 1, alongside their smoking status.

**Table 1:** Socio-demographic features of participants by smoking status.

	Socio-demographic variables	Whole sample n	Smoker	Non-smoker	p-value (Chi-square)
n		9,657	2,019	7,628	
Age band	16 to 24	865	11.9**%**	8.2%	
	25 to 34	1,306	18.8**%**	12.2%	
	35 to 44	1,730	20.5**%**	17.3%	
	45 to 54	1,731	18.5**%**	17.8%	p<0.001
	55 to 64	1,687	17.3**%**	17.5%	
	65 to 74	1,277	8.6**%**	14.5%	
	75 and over	1,051	4.4**%**	12.6%	
Gender	Male	4,304	45.9**%**	44.3%	
	Female	4,251	54.1%	55.7%	
Ethnicity	White British/other White	8,746	94.0**%**	89.9%	
	Mixed race	64	0.8**%**	0.6%	p = 0.20
	Asian-Indian	265	0.9**%**	3.2%	
	Asian-Pakistani & Bangladeshi	165	0.8**%**	2.0%	p<0.001
	Asian-Other	67	0.3**%**	0.8%	
	Black Caribbean	85	1.1**%**	0.8%	
	Black African	89	0.4**%**	1.1%	
	Other Black	4	0.1**%**	0.0%	
	Other ethnic group	149	1.4**%**	1.6%	
Deprivation (IMD)	1 *most deprived*	1,497	25.0**%**	13.0%	
	2	1,782	22.7**%**	17.3%	
	3	2,168	22.8**%**	22.4%	
	4	2,062	16.8**%**	22.6%	p<0.001
	5 *least deprived*	2,138	12.7**%**	24.7%	
Region	North East	990	11.5**%**	9.9%	
	North West	969	11.9**%**	9.5%	
	Yorkshire & the Humber	1,020	12.5**%**	10.1%	
	East Midlands	1,130	11.3**%**	11.8%	p<0.001
	West Midlands	873	8.2**%**	9.3%	
	East of England	1,033	10.6**%**	10.7%	
	London	761	7.8**%**	7.9%	
	South East Coast	896	8.9**%**	9.4%	
	South Central	966	6.9**%**	10.8%	
	South West	1,009	10.4**%**	10.5%	

### Smoking status

**Fig 1:**
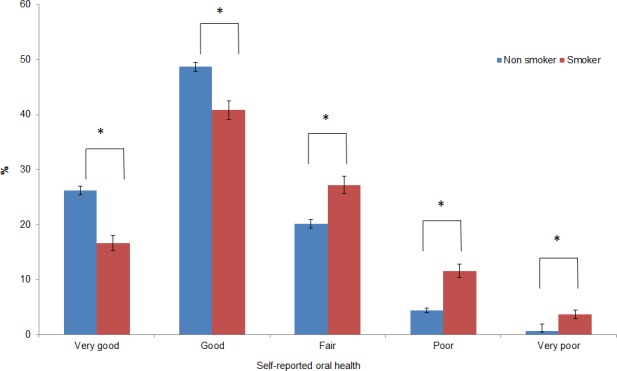
Self-reported oral health status by smoking status (percentage). (error bars indicated 95% confidence intervals) *Significance level 0.05 after Bonferroni correction.

Non-smokers were more likely to report very good or good self-reported oral health than smokers (75% versus 57%, Pearson’s Chi-square test, df = 1, p<0.001). Fig 1 illustrates the distribution of self-reported oral health status of these participants.

**Fig 2:**
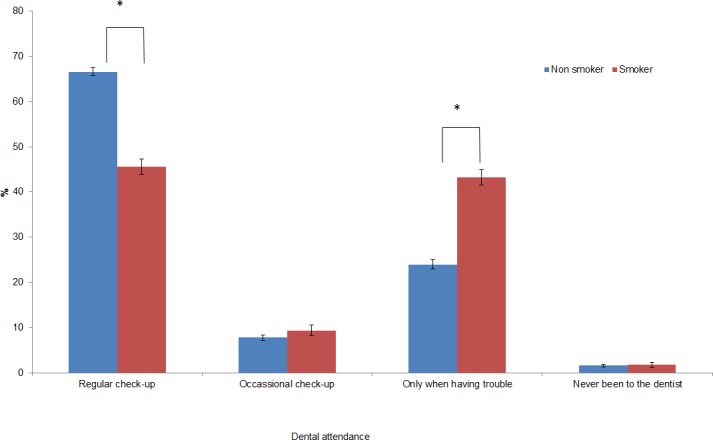
Dental attendance by smoking status (percentage). (error bars indicated 95% confidence intervals) *Significance level 0.05 after Bonferroni correction.

Dental attendance of non-smokers was statistically significantly different to that of smokers (Fig 2, Pearson’s Chi-square test, df = 3, p<0.001). Non-smokers were more likely to report attending for a regular check-up than smokers (67% vs 46%), while smokers were more likely to report attending when having symptoms (43% vs 24%).

The self-reported oral health status of smokers and non-smokers within each deprivation quintile also differed (Pearson’s Chi-square tests, df = 1, all p-values < 0.001). There was a significant difference in risk of having poor oral health for smokers compared with non-smokers for each IMD quintile. The overall risk for smokers reporting poor self-reported oral health was 17.5% higher than that of non-smokers. The odds for smokers reporting poor oral health was 2.21 times greater than non-smokers (Table 2). All deprivation quintiles indicated a significant high risk of reporting poor self-reported oral health if they were smokers when compared with non-smokers.

**Table 2:** Risks of self-reported poor oral health for smokers and non-smokers, their risk difference and odds ratio by deprivation quintile.

IMD quintile	Risk for smokers (%)	Risk for non-smokers (%)	Risk difference (%)	p-value	ORs (95% CIs)
1 *most deprived*	48.5	34.6	13.9	P<0.001	1.77 (1.42, 2.20)
2	46.2	25.1	21.1	P<0.001	2.56 (2.05, 3.20)
3	39.1	26.2	12.9	P<0.001	1.81 (1.46, 2.24)
4	38.3	22.5	15.8	P<0.001	2.14 (1.67, 2.74)
5 *least deprived*	36.7	21.5	15.2	P<0.001	2.12 (1.61, 2.80)
Overall	42.6	25.1	17.5	P<0.001	2.21 (2.00, 2.45)

Focusing on the participants who regularly attend the dentists for check-ups and those who only attend symptomatically (when they have problems) is outlined in Table 3 Overall, smokers were more than twice as likely as non-smokers to attend the dentist symptomatically (OR = 2.63). Smokers were significantly more likely to report attending symptomatically in all deprivation quintiles when compared with non-smokers (Table 2, Person’s Chi-square tests, df = 1, all p-values < 0.001).

**Table 3:** Risk of symptomatic dental attendance for smokers compared with non-smokers, their risk difference and odds ratio by deprivation quintile.

IMD 2010 quintile	Risk for smokers (%)	Risk for non-smokers (%)	Risk difference (%)	p-value	ORs (95% CIs)
1 *most deprived*	63.2	46.3	16.9	P<0.001	1.99 (1.57, 2.52)
2	51.3	34.9	16.4	P<0.001	1.96 (1.56, 2.47)
3	43.0	25.1	17.9	P<0.001	2.25 (1.79, 2.83)
4	43.2	19.9	13.3	P<0.001	3.07 (2.37, 3.96)
5 *least deprived*	33.6	18.0	15.6	P<0.001	2.03 (1.71, 3.12)
Overall	48.6	26.5	21.1	P<0.001	2.63 (2.36, 2.93)

### Predictors of poor self-reported oral health status and symptomatic dental attendance

A multivariate linear logistic regression model was used to assess the risk factors for poor self-reported oral health and symptomatic dental attendance, assuming independent sampling using complete-case analysis and weighted according to the survey weight provided in the ADHS dataset. The dependent variable was ‘fair/poor/very poor (poor) self-reported oral health status’ and ‘only when having problem’ in the dental attendance pattern, and the independent variables were listed in column 1 along with the reference case (as denoted by this symbol: †) (Table 4). Full models including interaction terms between independent predictors, such as interaction between deprivation index and smoking status, had been considered, but no significance of interactions are detected in both models. For example, the effect of smoking status on poor dental health is independent of deprivation quintile and so the original multivariate linear models was used. Multicollinearities within both models were tested and all variables showed low correlation measured by variance inflation factor VIF (VIF: 1 means low or no correlation of variables; VIF >5 indicates high multicollinearity) [18]. Both self-reported oral health status model and dental attendance model the VIF was around one which indicated that we do not have multicollinearity issue in our models (Oral health model: VIF_IMD_ = 1.33, VIF_Gender_ = 1.01, VIF_Ethnicity_ = 1.41, VIF_Age_ = 1.15, VIF_Smoking status_ = 1.09 and VIF_Region_ = 1.50; Dental attendance model: VIF_IMD_ = 1.27, VIF_Gender_ = 1.02, VIF_ethnicity_ = 1.36, VIF_Age_ = 1.16, VIF_Smoking status_ = 1.10 and VIF_Region_ = 1.43. There were 13 missing entries for participant’s ethnicity out of 9657, but coefficients obtained in the multiple logistic regression models for both self-reported oral health and dental attendance were very similar to the original outputs (all have the same value to 2 decimals), after multiple imputation.

**Table 4:** Risk factors for poor self-reported oral health status -multivariate logistic regression.

	Risk factor	p-value	ORs (exp(B)) to have poor oral health compared with reference category (95% Confidence interval)
Age band	16 to 24^†^		
	25 to 34	p<0.001	1.54 (1.24, 19.1) +++
	35 to 44	p<0.001	1.62 (1.30, 2.00)+++
	45 to 54	p<0.001	2.25 (1.81, 2.79)+++
	55 to 64	p<0.001	2.20 (1.78, 2.73)+++
	65 to 74	p<0.001	1.72 (1.36, 2.17)+++
	75 and over	p<0.001	2.10 (1.66, 2.65)+++
Gender	Male^†^		
	Female	p<0.001	0.82 (0.74, 0.90)+++
Ethnicity	White British/other White^†^		
	Mixed race	p = 0.05	0.49 (0.24, 1.00)
	Asian-Indian	p = 0.17	0.79 (0.56–1.10)
	Asian-Pakistani & Bangladeshi	p<0.001	2.16(1.52–3.07)+++
	Asian-Other	p = 0.64	0.86(0.47,1.58)
	Black Caribbean	p = 0.88	1.04 (0.61, 1.77)
	Black African	p = 0.46	0.82 (0.47, 1.42)
	Other Black	p = 0.55	0.50 (0.05, 5.02)
	Other ethnic group	p = 0.24	0.81 (0.53, 1.25)
Deprivation (IMD)	1 *most deprived*^†^		
	2	p<0.001	0.68 (0.57, 0.82)+++
	3	p<0.001	0.64 (0.54, 0.77)+++
	4	p<0.001	0.55 (0.46, 0.65) +++
	5 *least deprived*	p<0.001	0.51 (0.42, 0.60)+++
Smoke	No^†^		
	Yes	p<0.001	**2.29** (2.02, 2.55) +++
Region	North East^†^		
	North West	p = 0.25	1.14 (0.92, 1.41)
	Yorkshire & the Humber	p = 0.10	1.20 (0.97, 1.49)
	East Midlands	p = 0.91	0.99 (0.80, 1.23)
	West Midlands	p = 0.17	0.85 (0.67, 1.08)
	East of England	p = 0.02	1.30 (1.05, 1.61)+
	London	p = 0.006	1.39 (1.10, 1.76) ++
	South East Coast	p = 0.03	1.28 (1.03, 1.59)++
	South Central	p = 0.05	1.26 (0.99, 1.59)
	South West	p = 0.008	1.34 (1.08, 1.66)++

†reference category

Significance levels:

+++ <0.001

++ <0.01

+ <0.05

#### Predictors of poor self-reported oral health

Table 4 shows the coefficient of each predictor in the logistic regression model of poor self-reported oral health as the outcome. Every categorical predictor is significant. The largest odds ratio to have poor oral health is smoker compared with non-smoker (OR = 2.29), and the second largest odds ratio is to be Asian Pakistani & Bangladeshi compared with being White British (OR = 2.16), when controlling other risk factors. By age band, older people were more likely to have poor self-reported oral health compared with younger participants, and females were less likely to have self-reported poor oral health. IMD showed that the higher socio-economic class was, the less likely they would self-report poor oral health. Compared with North East, people in London and the south are more likely to self-report poor oral health.

#### Predictors of symptomatic dental attendance

In order to assess the behaviour difference between smokers and non-smokers in terms of their dental attendance pattern, we focused on the participants who attend dentist regularly (62.2% of the total responses) and those who only attend symptomatically (28.0%) (these two types of attendance make up over 90% of the total sampled subjects). Table 5 shows predictors of symptomatic attendance using a multivariate logistic regression model. Again, every categorical predictor is significant. The largest odds ratio to have symptomatic dental attendance is ethnicity (Asian-other, Asian-Indian and Black Africa compared with White British). Smokers compared with non-smokers had an odds ratio of 2.27 for symptomatic dental attendance. By age, those considered ‘middle aged’ were more likely to attend dental practice regularly, while the oldest participants (75 and over) and the younger group (25–34) were more likely to go to dental practice when they had symptoms (16–24 were the reference age group). Women are less likely to attend dental care symptomatically. IMD also showed those in higher socio-economic groupings were more likely to attend on a regular basis. Compared with North East, those from London and South East Coast were more likely to go to dentist when they had problems.

**Table 5:** Risk factors for symptomatic dental attendance versus regular check-up -multivariate logistic regression.

	Risk factor	p-value	OR (exp(B)) to have poor oral health compared with the reference category (95% CI)
Age band	16 to 24^†^		
	25 to 34	p = 0.04	1.24 (1.01, 1.50)+
	35 to 44	p<0.001	0.72 (0.59, 0.87)+++
	45 to 54	p<0.001	0.57 (0.47, 0.69)+++
	55 to 64	p<0.001	0.53 (0.44, 0.65)+++
	65 to 74	p<0.001	0.66 (0.55, 0.81)+++
	75 and over	p<0.001	1.56 (1.26, 1.95)+++
Gender	Male^†^		
	Female	p<0.001	0.58 (0.53, 0.64)+++
Ethnicity	White British/other White^†^		
	Mixed race	p = 0.13	1.52 (0.90, 2.58)
	Asian-Indian	p<0.001	**3.42** (2.50, 4.86)+++
	Asian-Pakistani & Bangladeshi	p<0.001	2.76 (1.87, 4.10)+++
	Asian-Other	p<0.001	**3.75** (2.12, 6.61)+++
	Black Caribbean	p = 0.44	1.21(0.74, 1.97)
	Black African	p = 0.002	**2.67** (1.45, 4.89)++
	Other Black	p = 0.94	0.93(0.14, 6.06)
	Other ethnic group	p = 0.008	1.78 (1.16, 2.75)++
Deprivation (IMD)	1 *most deprived*^†^		
	2	p<0.001	0.66 (0.56, 0.79) +++
	3	p<0.001	0.48 (0.41, 0.56)+++
	4	p<0.001	0.39 (0.33, 0.47)+++
	5 *least deprived*	p<0.001	0.35 (0.29, 0.42)+++
Smoke	No^†^		
	Yes	p<0.001	2.27 (2.02, 2.55)+++
Region	North East^†^		
	North West	p = 0.35	1.11 (0.89, 1.37)
	Yorkshire & the Humber	p = 0.47	1.08 (0.87, 1.34)
	East Midlands	p = 0.48	0.93 (0.74, 1.15)
	West Midlands	p = 0.40	1.09 (0.88, 1.36)
	East of England	p = 0.17	1.16 (0.94, 1.44)
	London	p<0.001	1.75 (1.38, 2.21) +++
	South East Coast	p = 0.006	1.35 (1.09, 1.67)++
	South Central	p = 0.69	1.05 (0.83, 1.33)
	South West	p = 0.20	1.15 (0.93, 1.43)

†reference category

Significance levels:

+++ <0.001

++ <0.01

+ <0.05

## Discussion

This study is the first to examine the self-reported oral health status and dental attendance of smokers and non-smokers in England. The study explored if a relationship between perceived oral health status and dental attendance of smokers noted within a previous study at a regional level [15] were evident nationally. The present study’s findings are broadly consistent with those of the regional study [15] which identified that being a smoker best predicted self-reported poorer oral health and symptomatic dental attendance. A strength of the current paper that it has built on localised findings from Yorkshire and the Humber, UK and has replicated some of these findings at a national level. This begins to construct an evidence base regarding smokers’ dental attendance and self-reported oral heath differing from those who do not smoke. Another strength of the present analysis is it uses a national dataset which has been standardised and validated since 1968 [16]. The Adult Dental Health Survey dataset is available centrally by registered users; this adds to the credibility to the present research and strengthens the external validity.

The dataset used was the Adult Dental Health Survey 2009 which reported a smoking prevalence of 21% of the respondents, which this is consistent with another UK lifestyle survey [20]. The South Central region had the lowest smoking prevalence and the North West the highest, which correlates with the deprivation status of each area [17]. The existence of such a correlation has been repeatedly reported with higher smoking prevalence in areas of greater disadvantage [21–23]. One surprising finding was the perception of poor oral health in the South West and South East Coast as this is an area of lower deprivation and therefore we would expect a higher self-perceived oral health. The reason for this finding is unclear, it may be related to how different groups in different areas of the country perceive and assess oral health, which has been identified as an under-researched area [24].

Overall smokers were more than twice as likely to report poor oral health compared with non-smokers. Participants in the least deprived areas who smoked were 1.7 times more likely to report poor oral health than non-smokers. Irrespective of deprivation status, those who smoked were more likely to report poor oral health than non-smokers. Logistic regression showed that smoking status yields the largest odds ratio (2.27) for predicting self-reported oral health status. Millar and Locker [25] reported similar findings, they found smokers were more likely to report oral pain than never smokers within a multivariate logistic model controlling for gender, age, household income, education and dental insurance. They found that current smokers and former smokers had higher odds of oral–facial pain than never smokers. An American study which analysed a national dataset found that current cigarette smokers were more likely to have higher levels of perceived dental needs when compared with non-smokers [26]. The importance of these findings extend to those delivering dental health services, those developing public health policies and those researching such issues to inform the evidence base of what works. Understanding the predictors to self-perceived dental need and self-reported oral health status (how people feel about their mouths) may improve our understanding of what encourages people to seek dental care.

Jorm and co-workers [27] reported that after adjusting for access and health-related factors, smokers were less likely to claim Medicare benefit, use primary care services (if there were associated costs) and use preventive services compared with non-smokers. Given smokers’ risk of periodontal disease, higher rates of oral cancer and lower preventive dental attendance there seems to be a public health challenge. More research is required to identify the best way to reach this group of the population to ensure they have the advice and access to cessation services necessary to reduce their risk from smoking. The challenge of the ‘inverse care law’ [28] where individuals with the greatest health inequalities (need) have the lowest access services has been further developed by assessing access to preventative services and care. The ‘inverse prevention law’ focuses on preventative service uptake by those in most need and follows the same principle: those who could benefit most from preventive interventions are least likely to receive them thus increasing health inequalities [29].

There was variation found in the perceived oral health and dental attendance in different ethnic groups. Asian-Pakistani & Bangladeshi participants were more likely to report poor oral health compared with participants from other ethnic groups and were more likely to report symptomatic attendance. The importance of ethnicity related to oral health has been hotly debated. A recent model of oral inequalities suggest ethnicity is only one factor contributing to oral health inequalities [30] and empirical evidence suggests much of this is socioeconomically driven [31]. Highlighting ethnicity as the sole predictor of self-reported oral health status and dental attendance may distract from the root causes of inequalities and risk stigmatising some groups [30].

The findings of this analysis and the regional study published in 2013 [15] have implications for dental teams’ ability to access smokers and therefore any effect they might have on helping smokers quit. Smokers are more likely to attend symptomatically and opportunities for smoking cessation interventions by the dental team may therefore be limited or unwelcome especially if patients are seeking pain relief. In addition, smokers perceived their oral health to be poorer than non-smokers and therefore support, guidance and clinical interventions necessary to improve the health of their mouths may be challenging to deliver if they only attend when having problems with their mouths. Oral health messages are more likely to have impact and be effective if part of continual care, are communicated clearly and are tailored to the patient [32]. Working with patients in this way builds familiarity and trust and supports a tailored prevention approach which improves the effectiveness of preventative advice [32].

It is essential to consider how policy currently supports smokers within the dental setting. There have been two Public Health England guidance documents produced in 2014: Smokfree and Smiling: Helping dental patients quit tobacco (Second Edition) [11] and Delivering Better Oral Health: An evidence-based toolkit for prevention (Third Edition) [33] which are focussed on supporting dental teams, commissioners and providers of smoking cessation services and dental educators to work with patients to quit tobacco. It is recognised that dental teams have crucial role to play in advising patients of the risks to their oral health and signposting to local cessation services [12, 34]. Policy and guidance has also extended to local authorities who hold budgets to improve oral health locally. The guidance for local authorities on improving oral health: commissioning better oral health for children and young people [35] provides the evidence and tools for local authorities to review and evaluate existing oral health improvement programmes and consider future commissioning intentions to provide an evidence-informed approach with examples of good practice. Tobacco is considered within this document as a significant public health issue and requires consideration of what interventions can support people to quit and improve their oral and general health. Beyond individualised support from dental teams and community based actions from local authorities are by those who design and commission dental services. Within England, new primary dental care contracts prototypes are being developed. The models are looking at remuneration of treatment, preventative care and advice. Smoking cessation, is of course part of the advice that dental teams have a key role to provide to their patients [36].

The importance of continued “upstream” population approaches outside of the clinical environment are essential to consider given the limitation dental teams may face attempting to reach smokers. Although the impact dental teams can have on smoking cessation has been demonstrated [12], the present findings suggest that contact with smokers within a dental setting may be limited as they are less likely to attend regularly and more likely to be symptomatic. Smoking not only increases the risk of oral cancer and periodontal disease but also stroke and heart disease [37], a common risk factor approach is necessary to target those at increased risk of a multitude of conditions and diseases and should be utilised across the spectrum health practitioners. If this model is applied, a joined up approach to the prevention of ill health could be achieved. The common risk factor approach focuses on healthy choices that not only impact on oral health but general health; giving health and social care workers multiple opportunities to work with smokers when they present themselves to ‘make every contact count’ [38].

One limitation within the analysis should be raised when considering the Index of Multiple Deprivation (IMD 2010). IMD was used to estimate deprivation and as a composite index incorporates a range of data from a neighbourhood of up to 6000 households. This index does not necessarily indicate the level of deprivation experienced by each participant and consequently, participants in this study may or may not experience the estimated deprivation for that area. The indices are a measure of area deprivation, within this analysis deprivation scores were allocated into quintiles (least, less, average, more and most deprived) as this is commonly used in such a way within the field of health and social care [39]. Within the present research retrospective data was used, it is difficult to be wholly confident that respondents categorised into deprivation quintiles do in fact experience the level of deprivation assigned, caution therefore should be applied when interpreting results. Future research could consider collecting prospective data and therefore establish the deprivation status of each participant more confidently.

The oral health status and dental attendance data were self-reported, participants were volunteers and this increases the risk that “self-selecting” samples will not be representative of the source population [40]. As smoking prevalence was similar to another large UK survey [20], any sampling bias is likely to be similar, but the impact of such bias is unclear. However, the use of self-reported measures has been validated as robust measure when assessing the needs at a population level but there are limitations [41]. For example, self-reported measures could under or overestimate treatment need. Within the present study, unpicking variables related to dental attendance and perceptions of oral health status were necessary as personally derived oral health status is one factor that stimulates an individual to seek care and advice [42].

## Conclusions

This paper has reported the self-reported oral health status and dental attendance of smokers and non-smokers in England. Smokers perceived they had poorer oral health and were more likely to attend symptomatically than non-smokers even when considering the deprivation status of the participant. The present study’s findings are consistent with earlier sub-national research. These findings should be considered when planning early diagnosis and management strategies for smoking-related conditions, considering the potential impact dental teams might have on smoking rates.
